# Supplementary material for: Racial disparities in post-transplant stroke and mortality following stroke in adult cardiac transplant recipients in the United States
Source: PLoS One. 2023 Feb 16;18(2):e0268275. doi: 10.1371/journal.pone.0268275 (PMC9934340; doi:10.1371/journal.pone.0268275)
Supplement: Attachment — Submitted filename: Liou_RacialDisparities_plos_one_reviewer_comments_v3.docx [file pone.0268275.s001.docx]

**Reviewer #1**

In page 6 the authors state: “We defined post-transplant stroke as a stroke…... We adjusted for clinical and demographic factors identified by previous studies examining the association between race and mortality.” Then they go on to list 20 variables that they adjust for. In stroke cohorts baseline NIHSS and age were the major determinants of outcomes (See Uchino, Billheimer, Cramer Stroke 2001; Mandava, Kent 2009). Baseline NIHSS was the sole determinant of outcome for individual patients as well (Adams et al, Neurology 1999). Just because there were 900 patients having 20 variables does not give a reader added confidence in the study.

We thank the reviewer for this comment. Since the SRTR is an administrative transplant dataset rather than intended for studying stroke patients, we cannot quantify the severity of a stroke using the NIHSS scale. We now acknowledge this limitation in the analysis of post-stroke mortality, stating that “We did not have detailed information on the clinical presentation and acute management of stroke nor NIHSS as SRTR is not strictly a stroke cohort dataset, but rather an administrative transplant dataset.” (page 15). We also note that we adjusted for well-established risk factors for stroke and mortality as in previously published studies using SRTR data.

Registry data sets are notoriously unreliable (Willis et al, International Journal for Quality in Health Care, 2011), accentuating the already uncertain nature of heterogenous disease such as stroke. Now adding imputation does not give the reader confidence regarding generalizability of the data. Garg in Acta Neurologica Scandinavica, 2022 has suggested that fragility index be used to assess the value of imputation.

We thank the reviewer for this comment. Regarding the heterogeneity of stroke diagnoses, we agree that it would be preferable to have data on stroke subtype and severity. Nevertheless, the SRTR data has previously been used in published literature and maintains the highest quality of registry data, linking records with OPTN, CMS, and NTIS’s Death Master File and includes monthly updates. Therefore, despite its limitations, this resource allows us to explore important questions, as noted by Massie and colleagues (<https://www.ncbi.nlm.nih.gov/pmc/articles/PMC4387865/>).

Regarding our use of multiple imputation using chained equations (MICE), we followed standard practice to address missingness to reduce rather than to enhance generalizability. The reviewer cites an article on the concerns about imputing outcome data. We, therefore, now clarify on page 7 that we imputed missing covariate data but not missing exposure or outcome data.

That there are geographical disparities of stroke care in the US is well known. And that the authors cited Howard et al, Annals of Neurology 2011 is noted. However, there are other studies pointing to race based differential nature of response to thrombolytic treated of patients suffering a stroke (Mandava et al, Stroke 2013). However, a study published the same year (Mishra et al, International Journal of Stroke, 2013) did not find differences in response to thrombolytic treatment. Xian et al, Annals of Internal Medicine 2011 report that blacks had lower mortality compared to whites. Xian et al and many others have discussed end of life care of black patients compared to white patients is different. Would the authors attempt to explain the results of worse mortality in black heart transplant patients. Could this be due to geographic difference in care provided to blacks, response to treatment of black patients or choice of end of life care meted to blacks.

We thank the reviewer for this comment. On page 12, we now clarify that our primary analysis focused on the recent era of 2010-2018, in which we found that Black patients had slightly better survival than white patients, which may reflect overall protocol improvements for heart transplant recipients irrespective of race, such as advancements in surgical techniques and immediate postoperative care. As a secondary analysis, we reported the finding of worse mortality in black heart transplant recipients in our full cohort (1987-2018), concordant with previous studies on race and post-transplant mortality. On page 12, we suggest that worse mortality outcomes are likely being driven by previously persistent racial disparities in the earliest transplantation era. We cite studies such as Liu et. al. Transplantation 2011 and Kilic et. al. Transplantation 2015 which list potential mechanisms for these disparities, such as disparities in access to medical therapy before and after transplantation, socioeconomic status, dietary habits, pharmacokinetics, and genetic expression and polymorphisms to adversely affect Black patients in previous transplantation eras. Based on the reviewer’s comment, we now add this may also be due to differences in end-of-life care and cite Xian et. al. 2011.

We thank the reviewer for the suggestion to examine geographic differences. We now perform a sensitivity analysis including transplant center as a stratifying variable. This allows us to enable flexible estimation of baseline hazards per transplant center and tightly control for not only geographical variability, but also other potential unmeasured confounding related to care practices, physicians, and transplant center characteristics. The results of this analyses were not materially different from our main results for overall mortality (HR = 1.14, 95% CI: 0.87 – 1.48), 6-month mortality (HR = 1.01, 95% CI: 0.73, 1.40), and mortality beyond 6 months (HR = 1.49, 95% CI = 0.92, 2.39). We do not have a sufficient number of deaths per transplant center to conduct subgroup analyses by center.

**Reviewer #2**

Were patients from other racial groups (other than Black and White) included? If not a description of how many patients excluded and why will be useful. Also, when reviewing table 1 some numbers seem inaccurate; it is evident that transplant volume has grown in most recent period therefore, period of 2010 to 2018 (9 years) should have similar number of transplants performed compared to 1987-1999 (12 year period). In the table 1987-99 has more transplant performed which should carefully re-analyzed.

We thank the reviewer for this point of clarification. In the Methods section (page 5), we now add that “5,838 adults of other reported racial and ethnic backgrounds were not included in this study cohort to focus the scope of this research study extending previous literature investigating disparities in outcomes between Black and white transplant recipients.”

We also thank the reviewer for carefully evaluating Table 1. We verified our numbers of transplants filtering the dataset to transplantations performed between January 1, 1987 and December 31, 2021, and the values seem correct. We plotted the number of unique patient records for heart transplants on each day between 1987 and 1999, and there was no evidence of any unusual patterns that would suggest a data coding error in transplantation date.

What was timing/definition of post-transplant stroke? Was this immediately post-transplant (within 30 days) or early (within 1-year)? This is very critical information. Must be elaborated.

We thank the reviewer for making this important point. On page 6, we now add that “We defined post-transplant stroke as a stroke occurring within 30 days after the transplant procedure”.

Did the authors adjust for PA pressures, renal function (creatinine), Bilirubin, inotrope support?

Did you consider pre-transplant stroke or history of cerebrovascular disease as a risk factor? It would be a valuable edition.

We thank the reviewer for this valuable suggestion We adjusted for PA pressures and inotrope. We now additionally adjusted for the most recent recorded bilirubin, creatinine levels, pre-transplant stroke and history of cerebrovascular disease. The conclusions remain the same from the refit models, but the addition of these confounders attenuated the associations slightly. On page 6, we now state that we adjust for these confounders.

How were PRA handled? Multiple values available. Were most recent ones used?

We thank the reviewer for this question. We used a combination of both the most recent calculated panel reactive antibody measurement (REC_CPRA) and the most recent observe panel reactive antibody measurement (REC_PRA_MOST_RECENT) where if REC_CPRA was missing, we used the REC_PRA_MOST_RECENT value instead. This handling of PRA was performed exactly as in Salia et. al. AJT 2022.

Based on your work it is evident that some variables have extensive missing values. Did you evaluate if these values were missing for a specific time frame? I.e. missing for earlier patients (prior to 1999). This may help you to limit the cohort to certain time period where you do not have to deal with extensive missing values. Also, consider the fact that most transplant have occurred in last 2 decades with more reliable and complete data available.

We thank the reviewer for this insightful question. For certain variables that had particularly high proportions of missingness such as education, insurance, smoking status, functional status, and VAD, they were missing predominantly from the earliest transplant era (prior to 2000). Other variables such as the setting of follow-up care and hypertension had similar missing rates in each transplant era.

To investigate whether the extent of missingness is related to the modeled estimates, we compared the hazard ratios estimated for the full cohort 1987-2018 to those from a restricted cohort of 2000-2018 for each transplantation era, adjusting for the set of confounders that we used in our original manuscript. As an example, for the beyond 6-month hazard ratios presented below, we see that they do not substantially change, which suggests to us the extent of missingness and our subsequent multiple imputation approach is not substantially affecting our results. We also emphasize that we use time in our multiple imputation approach.

We, therefore, decided to keep our cohort to 1987-2018 for the primary purpose of illustrating changing disparities over time across a broader time range of transplantations performed in the US. We acknowledge that data quality may be worse in the earliest era. On page 15, we note that “For some of the variables with a high proportion of missingness, we observed that the missingness was coming from the 1987-1999 transplantation era. However, the results and conclusions do not change even after running a sensitivity analysis in which we restricted the cohort to 2000-2018. Furthermore, keeping the cohort timeframe between 1987 and 2018 enables us to observe broader patterns in how racial differences in post-transplant mortality outcomes for post-transplant stroke survivors have changed over time”. If the editor feels strongly, we’d be happy to include what we’ve shown as a sensitivity analysis in an online supplement.

| Transplantation Era | Cohort of 1987-2018 HR (95% CI) | Cohort of 2000-2018 HR (95% CI) |
| --- | --- | --- |
| Jan 1, 1987 –  Dec 31, 1999 | 2.32 (1.19, 4.50) | - |
| Jan 1, 2000 –  Dec 31, 2009 | 2.03 (1.02, 4.01) | 2.11 (1.04, 4.28) |
| Jan 1, 2010 –  Dec 31, 2018 | 0.86 (0.43, 1.72) | 0.95 (0.49, 1.89) |

Although authors described the survival based on 3 time periods, they have not provided differences in transplant population between these time periods. It would be important to know population risk changes over time and its impact on survival outcomes.

We thank the reviewer for this suggestion. On page 24, we now include a table describing the differences in transplantation population between these time periods. We now explain that “Baseline characteristics for Black and white post-transplant survivors across the three transplantation eras are summarized in Table 2. Over time, there is a tendency of a greater proportion of Black females and a greater proportion of Black recipients who have private insurance, obesity, hypertension, and high PRA values receiving heart transplants.”
